# Absolute Quantitative Targeted Monitoring of Potential Plasma Protein Biomarkers: A Pilot Study on Healthy Individuals

**DOI:** 10.3390/biomedicines12102403

**Published:** 2024-10-21

**Authors:** Alexey S. Kononikhin, Natalia L. Starodubtseva, Alexander G. Brzhozovskiy, Alisa O. Tokareva, Daria N. Kashirina, Natalia V. Zakharova, Anna E. Bugrova, Maria I. Indeykina, Liudmila Kh. Pastushkova, Irina M. Larina, Vladimir A. Mitkevich, Alexander A. Makarov, Evgeny N. Nikolaev

**Affiliations:** 1 Project Center of Advanced Mass Spectrometry Technologies, 121205 Moscow, Russia; agb.imbp@gmail.com; 2 Institute of Biomedical Problems, Russian Federation State Scientific Research Center, Russian Academy of Sciences, 123007 Moscow, Russia; daryakudryavtseva@mail.ru (D.N.K.); lpastushkova@mail.ru (L.K.P.); irina.larina@gmail.com (I.M.L.); 3 V.I. Kulakov National Medical Research Center of Obstetrics, Gynecology, and Perinatology, Ministry of Health of Russia, 117997 Moscow, Russia; n_starodubtseva@oparina4.ru (N.L.S.); alisa.tokareva@phystech.edu (A.O.T.); anna.bugrova@gmail.com (A.E.B.); 4 Moscow Center for Advanced Studies, 123592 Moscow, Russia; 5 Emanuel Institute of Biochemical Physics, Russian Academy of Sciences, 119334 Moscow, Russia; nvzakharova@yandex.ru (N.V.Z.); mariind@yandex.ru (M.I.I.); 6 Engelhardt Institute of Molecular Biology, Russian Academy of Science, 119991 Moscow, Russia; mitkevich@gmail.com (V.A.M.); aamakarov@eimb.ru (A.A.M.)

**Keywords:** proteomics, blood, plasma, healthy volunteers, biomarkers, MRM, mass spectrometry

## Abstract

Background/Objectives: The development of blood tests for the early detection of individual predisposition to socially significant diseases remains a pressing issue. Methods: In this pilot study, multiple reaction monitoring mass spectrometry (MRM-MS) with a BAK-270 assay was applied for protein concentrations analysis in blood plasma from 21 healthy volunteers of the European cohort. Results: The levels of 138 plasma proteins were reliably and precisely quantified in no less than 50% of samples. The quantified proteins included 66 FDA-approved markers of cardiovascular diseases (CVD), and other potential biomarkers of pathologies such as cancer, diabetes mellitus, and Alzheimer’s disease. The analysis of individual variations of the plasma proteins revealed significant differences between the male (11) and female (10) groups. In total, fifteen proteins had a significantly different concentration in plasma; this included four proteins that exhibited changes greater than ±1.5-fold, three proteins (RBP4, APCS, and TTR) with higher levels in males, and one (SHBG) elevated in females. The obtained results demonstrated considerable agreement with the data collected from 20 samples of a North American cohort, which were analyzed with the similar MRM assay. The most significant differences between the cohorts of the two continents were observed in the level of 42 plasma proteins (including 24 FDA markers), of which 17 proteins showed a ≥1.5-fold change, and included proteins increased in North Americans (APOB, CRTAC1, C1QB, C1QC, C9, CRP, HP, IGHG1, IGKV4-1, SERPING1, RBP4, and AZGP1), as well as those elevated in Europeans (APOF, CD5L, HBG2, SELPLG, and TNA). Conclusions: The results suggest a different contribution of specific (patho)physiological pathways (e.g., immune system and blood coagulation) to the development of socially significant diseases in Europeans and North Americans, and they should be taken into account when refining diagnostic panels.

## 1. Introduction

Human plasma serves as a major source of protein biomarkers and ranks among the most studied clinical specimens due to its collection through minimally invasive techniques [1]. Despite a huge amount of research, the clinically important properties of the plasma proteome remain largely unexplored. In particular, the inherent variability in plasma protein levels within the population, as well as differences due to factors such as sex, age, and ethnicity, require further clarification. Exploring these issues can contribute to solving the problem of early diagnosis of various diseases. Information on multiple plasma protein markers is contained in several commercial databases, such as GOBIOM (https://gobiomdbplus.com/), Biomarkerbase (https://www.biomarkerbase.com/), or the Human Gene Mutation Database (http://www.hgmd.cf.ac.uk/ac/index.php, accessed on 28 May 2024). Additionally, detailed data on 142 protein markers can be accessed through freely available resources like MarkerDB (https://markerdb.ca) [2]. The actual number of described plasma biomarkers is much larger; the number of cardiovascular disease (CVD) markers alone already exceeds 150 [3]; and the use of aptamer-based proteomic profiling revealed 156 candidate biomarkers of CVD [4]. Particular plasma proteomic changes also have promising potential for early detection and continuous monitoring of other socially significant diseases, including cancer, diabetes mellitus, and Alzheimer’s disease [5,6,7].

However, before these biomarkers can be used in clinical diagnosis, they need to be analyzed for accuracy, reliability, and specificity. Additionally, when validating biomarkers, a question arises about the reproducibility of results in different laboratories and in different countries. It is also important to consider that variability in the levels of individual proteins can be associated not only with pathological abnormalities, but also with other characteristics of the studied cohorts, such as ethnicity and geography of residence [8,9].

Multiple reaction monitoring (MRM) is one of the most advanced tools of mass spectrometry (MS)-based proteomics that can be used for biomarker validation and for advanced comprehensive analysis to identify the early risk of developing cancer, CVD, and other socially significant diseases [10,11]. Recently, a panel of 270 human plasma proteins, including 61 FDA (U.S. Food and Drug Administration)-approved protein biomarkers [3], as well as other potential biomarkers of CVD, diabetes mellitus, cancer, neurodegenerative diseases (including AD), renal diseases, eye diseases, bone diseases, blood diseases, pregnancy complications, and autoimmune diseases, has been developed [9,12,13,14]. This panel showed high accuracy and reproducibility of the results, as well as the suitability of the selected peptides for reliable quantitative analysis of proteins in various samples [9]. It has been analyzed for interethnic and intersexual differences. It is important to note that in the study of the North American cohort, the overwhelming majority of proteins showed no significant differences between different ethnic groups, except for four proteins. The hemoglobin subunit alpha, apolipoprotein A-IV, and apolipoprotein C-III concentrations were found to be significantly higher in the Hispanic group than in the Black group, while the immunoglobulin heavy constant gamma 1 was significantly lower in the Hispanic group [9]. Some differences were observed among individuals of different sexes, as follows: CD5 antigen-like, sex hormone-binding globulin, fibronectin, and immunoglobulin heavy constant mu were significantly higher in females; and apolipoproteins A-IV and D were significantly higher in males [9].

Herein, to assess the variability of protein markers between cohorts from different continents, the panel of 270 proteins was applied for MRM analysis of plasma samples from healthy volunteers of a European cohort. A comparison of the current results with previous data suggests that the impact of specific (patho)physiological pathways in the development of socially significant diseases may differ between Europeans and North Americans. Overall, the data for the two cohorts are in good agreement, complement each other, and should be taken into account when refining diagnostic panels.

## 2. Materials and Methods

### 2.1. Object of Analysis

The study involved 10 healthy young women and 11 healthy men (aged 35 ± 6 years). All subjects are of European descent. All of the volunteers were found to be healthy by the medical expert commission. The study was conducted according to the guidelines of the Declaration of Helsinki and approved by the local Ethical Committee of the Institute of Biomedical Problems of the Russian Academy of Sciences (protocol No. 621, 8 August 2022).

### 2.2. Blood Collection and Sample Preparation

Blood samples (~6 mL per sample) from 21 healthy individuals were taken from a vein in the cubital fossa into Monovette tubes (SARSTEDT, Numbrecht, Germany) containing EDTA (K3). No protease inhibitors or antimicrobial agents were added. The samples were centrifuged for plasma separation (2000 rpm for 15 min, +4 °C) immediately after collection. The supernatant was frozen at −80 °C and was defrosted prior to sample preparation and LC-MS analysis.

The study was performed with non-depleted plasma samples using a BAK 270 assay kit (MRM Proteomics Inc., Montreal, QC, Canada) consisting of two synthetic peptide mixtures, as follows: one, containing unlabeled 270 matching (natural abundance) “light” peptides (NAT), which were used to prepare the calibration curves; and the second, containing 270 isotope-labeled standard (SIS) “heavy” peptides, which were spiked into each sample and served as internal standards for normalization [9]. Sample preparation was carried out according to the manufacturer’s protocol, using 10 μL of a plasma sample. Before trypsinolysis, the samples were denaturated and reduced by incubation with 9 M urea, 20 mM dithiothreitol, and 200 mM Tris × HCl (pH 8.0, +37 °C, 30 min). Next, proteins were alkylated by a 30 min incubation in the dark with 100 mM iodoacetamide. For trypsinolysis, the samples were diluted with 100 mM Tris × HCl (pH8.0) until there was <1 M urea; L-(tosylamido-2-phenyl) ethyl chloromethyl ketone (TPCK)-treated trypsin (Worthington, OH, USA) was added at a 20:1 (protein:enzyme, *w*/*w*) ratio; and the samples were incubated for 18 h at 37 °C. The reaction was quenched by acidifying the samples with formic acid (FA), reaching a final concentration of 1.0% (pH ≤ 2). The concentration of peptides in the resulting mixture was ~1 mg/mL [9]. After spiking with SIS peptides, the samples were cleaned up by solid-phase extraction (SPE) and reconstituted in 34 μL of 0.1% FA prior to LC-MS/MS analysis.

### 2.3. LC-MRM-MS Analysis

All samples were analyzed in duplicate by an HPLC-MS system consisting of an ExionLC™ UHPLC system (ThermoFisher Scientific, Waltham, MA, USA), coupled online to a SCIEX QTRAP 6500+ triple quadrupole mass spectrometer (SCIEX, Toronto, ON, Canada). LC-MS parameters, such as the LC gradient, and the MRM parameters (Q1 and MRM scans), were adapted and optimized based on the previous studies [9] (Supplementary Table S8).

The loaded sample volume was 10 μL per injection. HPLC separation was carried out using an Acquity UPLC Peptide BEH column (C18, 300 Å, 1.7 μm, 2.1 mm × 150 mm, 1/pg) (Waters, Milford, MA, USA) with gradient elution. Mobile phase A was 0.1% FA in water; mobile phase B was 0.1% FA in acetonitrile. LC separation was performed at a flow rate of 0.4 mL/min using a 53 min gradient from 2 to 45% of mobile phase B. Mass spectrometric measurements were carried out using the MRM acquisition method. The electrospray ionization (ESI) source settings were as follows: ion spray voltage, 4000 V; temperature, 450 °C; and ion source gas, 40 L/min.

The procedure for LC injection of samples, blanks, three levels of quality control (QC-A, QC-B, QC-C), and eight levels of calibration (A–H) are given in Supplementary Table S9.

Skyline Quantitative Analysis software (version 20.2.0.343, University of Washington) was used for quantitative analysis [15]. Calibration curves were generated using 1/(x × x)-weighted linear regression methods to calculate the peptide concentrations in the measured samples (fmol per 1 µL of plasma).

### 2.4. Standard and Quality Control Acceptance Criteria

This study was performed in accordance with the ICH guidelines on Bioanalytical Method Validation [9,16]. The calibration standards (eight levels, A–H) and QCs (three levels, QC-A, -B, and -C) were used to monitor the assay performance (Supplementary Tables S1 and S2).

The acceptance criteria for standards and QCs were accuracy and precision less than 20%, as well as a calculated concentration within ±20% of the theoretical value. Accuracy was calculated as the percent deviation (DEV) from the nominal value. Precision was obtained as √(mean square)/mean × 100%.

A standard curve was acceptable if at least 5 out of the 8 standards passed the acceptance criteria. For the experiment to succeed, at least 2/3 (66%) of all QC samples, and at least 90% of the peptide calibration curves, must be acceptable.

### 2.5. Statistical Methods

Unsupervised analysis was performed by hierarchical cluster analysis using Ward’s method. The clusters were merged by minimization of the intracluster variance increase, based on Manhattan distance, as follows: $\underset{i}{\sum }\left|x_{i}^{1}-x_{i}^{2}\right|$, where $x_{i}^{1}$ и $x_{i}^{2}$ are the *i*-coordinate values (autoscaled protein concentration) of sample 1 and sample 2, respectively. The statistical analysis was performed using a Mann–Whitney test with the threshold of significance of *p* < 0.01. Median fold changes were calculated for significantly different proteins by dividing median values. Analysis was performed using R-script 4.3.2.

A coefficient of variation (CV) for each protein in an individual sample was calculated as the ratio of the standard deviation of the calculated concentrations in parallel runs to their average concentration.

Protein–protein interactions were analyzed using the STRING database (accessed on 6 January 2023). Only associations with *p* < 0.05 were included in the final network. Protein categorical annotations were derived from Gene Ontology via the Swiss-Prot database.

## 3. Results

### 3.1. Human Plasma 270 Protein Assay Performance

Eight calibration standards (levels A–H) and three levels of quality controls (QC-A, -B, and -C) were injected three times to access assay performance. The QCs and standards’ accuracy and precision were calculated (Supplementary Tables S1 and S2).

All proteins exceeded 0.99 for calibration curve r^2^; among them, 238 were quantifiable in accordance to the ICH guidelines on Bioanalytical Method Validation [9,16], and acceptance criteria are described in Methods (Section 2.4). Among them, 71 proteins belonged to FDA-approved markers, and 50 were CVD markers (Supplementary Table S3).

For 192 (26, 12, and 8) proteins, A (B, C, and D), the calibration level was the lowest used for calibration curve calculation (Supplementary Tables S1 and S2). LLOQs were lower than the lowest calibration level for all proteins.

### 3.2. Individual Variations of the Plasma Proteins Studied

A total of 138 proteins with concentrations above the LLOQ were detected in at least 50% of the samples (Supplementary Tables S3–S5). The correlation between measured concentrations for two different peptides of the same protein was estimated for complement C4 (VGDTLNLNLR and VLSLAQEQVGGSPEK) and the insulin-like growth factor-binding protein complex acid labile subunit (LAELPADALGPLQR and NLIAAVAPGAFLGLK). In both cases, the correlation was 0.98.

**Figure 1:**
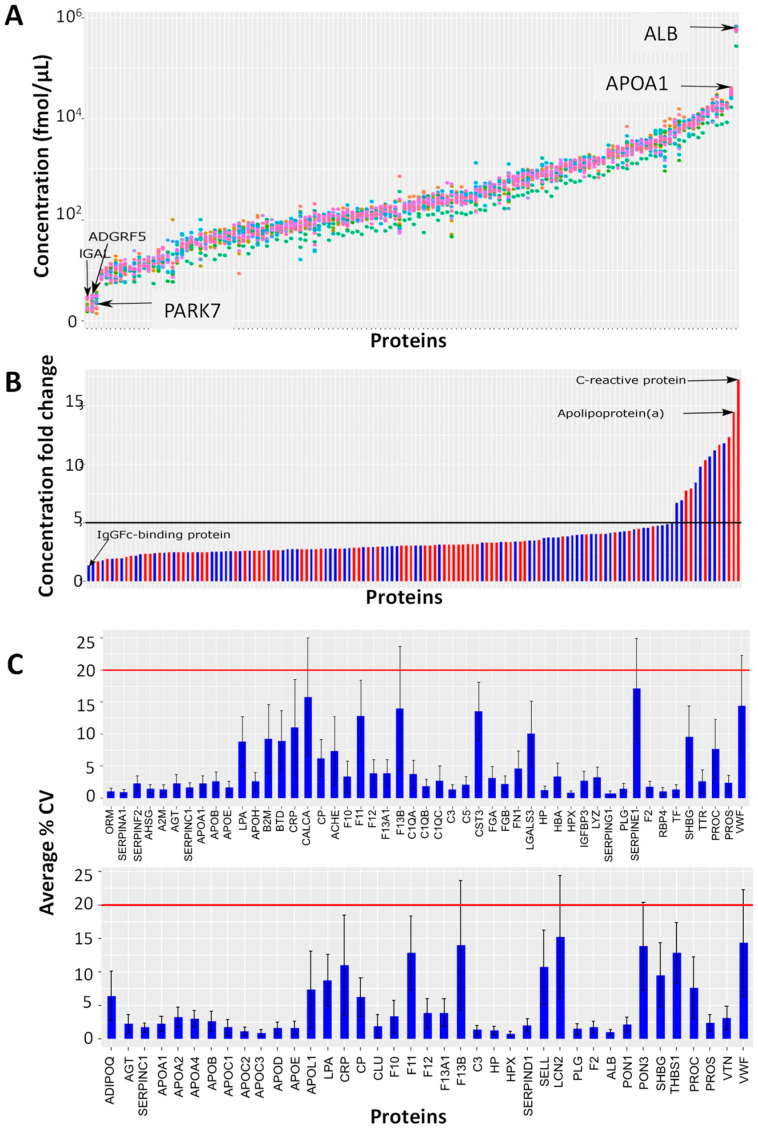
Results of data processing for 138 proteins reliably quantified in 21 plasma samples of European cohort. (**A**) Ranges of average protein concentrations; each sample is marked with a different color; proteins are arranged in order of increasing concentration. (**B**) The degree of variability in concentrations for each analyzed protein; proteins are arranged in order of increasing concentration variability; red color bars highlight FDA-approved and/or CVD markers. (**C**) Average % CV calculated for each FDA (upper plot) or CVD (lower plot) biomarker measured from all technical replicates.

Figure 1 shows the results of data processing for 138 proteins reliably quantified in 21 plasma samples. The range of measured concentrations in the same MS run covered five orders of magnitude (Figure 1A), as follows: the lowest average concentration threshold was determined for the neutrophil gelatinase-associated lipocalin (1.89 fmol/μL), and the maximum concentration was represented by serum albumin (577,475.4 fmol/μL). For most proteins, the ratio of maximum to minimum concentration was less than five (Figure 1B). IgGFc-binding protein (FCGBP) was the least variable protein, with a concentration fold change of 1.3. C-reactive protein and apoliprotein (a) were the most variable proteins, with concentration fold changes of 17.3 and 14.5, respectively (Supplementary Table S5). Notably, 49 proteins were FDA-approved markers and 39 proteins belonged to CVD markers (Supplementary Table S5, Figure 1C). For all of these proteins, the average % CV of variation was less than 20% (Figure 1C).

The concentrations of the remaining 100 proteins in the analyzed cohort were below the sensitivity range; however, their concentrations may significantly increase in certain pathologies.

### 3.3. Sex-Related Differences of the Plasma Proteins

**Figure 2:**
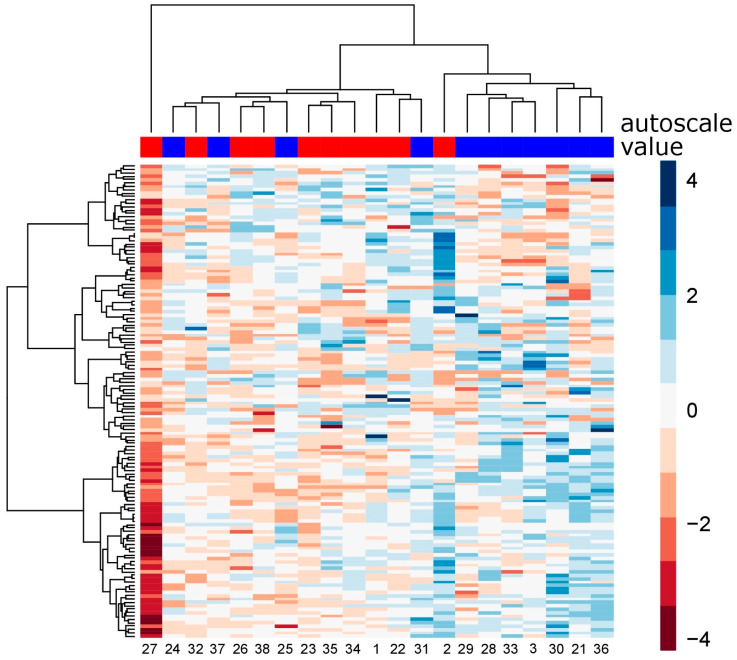
Hierarchical clustering of individual plasma samples and 138 quantified proteins, using Ward’s method and based on Manhattan distance. The upper bar shows whether the samples belong to the male (blue) or female (red) group.

Each sample had a unique fingerprint. At the same time, even for only 138 reliably quantified proteins, there were clear differences between the samples of males and females. (Figure 2). The most variable proteome profile was observed for one plasma sample from a female cohort member (#27), which was out of the main clusters (left side, Figure 2). This result is in agreement with proteome variability, which was discussed in a previous section (see Figure 1B). A comparison using a Mann–Whitney U-test revealed 15 proteins with a *p*-value < 0.01 (Table 1, Supplementary Table S6). However, only four proteins were out of a ±1.5-fold change, which are as follows: retinol-binding protein (RBP4), serum amyloid P-component (APCS), and transthyretin (TTR) were higher in males, while sex hormone-binding globulin (SHBG) had an increased concentration in female samples.

### 3.4. Comparison of Results for North American and European Cohorts

**Figure 3:**
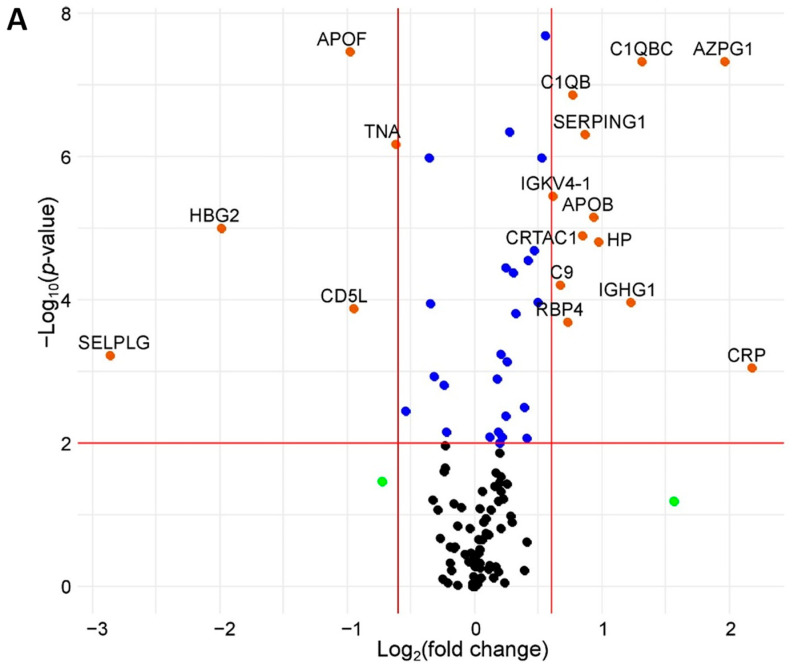

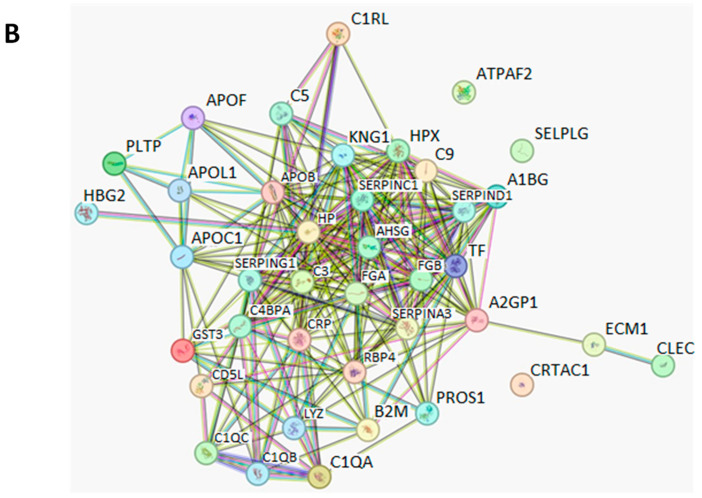
Quantified blood plasma proteins with significantly different concentrations between North American (n = 20, [9]) and European (n = 21, current study) cohorts. (**A**) Volcano plot: black dots indicate non-different proteins; green dots, proteins with ≥1.5-fold change; blue dots, proteins with *p* < 0.01; red dots, proteins with both ≥1.5-fold change and *p* < 0.01. (**B**) Protein–protein interaction (GO analysis).

The earlier study of plasma samples of the North American cohort [9] was performed with the same MRM approach, using the same procedure for sample preparation, included 20 participants (male/female, 10/10; Hispanic ethnic group, 60%, Black ethnic group, 35%; and Caucasian ethnicity, 5%), and reliably quantified 142 proteins, 127 of which were detected in >50% of samples, while 112 proteins matched the proteins quantified in the European cohort in the present study. The Mann–Whitney U-test identified 42 (37.5%) proteins whose levels turned out to be significantly different (*p*-value < 0.01) between the two studies, and 17 (15%) of them were different by ≥1.5 times (Figure 3A, Table 2); 24 of the proteins belong to FDA-approved and/or CVD markers. Figure 3B and Supplementary Table S7 show 81 molecular pathways, overrepresented by these proteins, mainly linked with the immune system and blood coagulation.

## 4. Discussion

Of the 270 analyzed target proteins, 138 were quantified in more than half of the samples, with performances generally comparable to those of the North American cohort study [9]. The quantified proteins included a number of potential biomarkers whose levels may be altered in CVD, cancer, AD, and other socially significant diseases. Importantly, these markers were detected with less than 20% SD between technical replicates, highlighting the fairly good quality and precision of the selected peptides, and demonstrating their suitability for reliable measurements and quantification in a variety of samples that can be easily transferred to clinical settings.

The ranges of measured concentrations of each specific protein were different (Figure 1B). The results for proteins with the greatest variability, C-reactive protein (17.3-fold difference) and apolipoprotein (a) (14.5), were the same as shown earlier [9], and confirm the high accuracy and reproducibility of the method in laboratories of different countries. At the same time, serum amyloid A-1, which earlier showed the greatest (69-fold) variability, turned out to be less variable in the current study (10.2-fold). Nevertheless, this protein was not included in the data presented, as it did not surpass one of the standard control acceptance criteria, as follows: only five out of nine QCs’ precision levels were below the 20% threshold. Seven FDA-approved and CVD markers (C-reactive protein, apolipoprotein (a), adiponectin, sex hormone-binding globulin, hemoglobin subunit alpha, transthyretin, and thrombospondin-1) revealed a considerable variability among the healthy volunteers. Some of the results obtained here are also in accordance with a SWATH-MS study [17], where 174 of 342 proteins showed more than a tenfold change between the extremes of the entire cohort; the standard deviation was minimal for antithrombin III and vitamin D-binding proteins; and the most variable proteins were apolipoprotein (a) and serum amyloid protein A-1. In general, proteins with high concentration variability in healthy individuals deserve special attention as potential diagnostic or prognostic targets. In this context, the use of MRM-MS-based methods for their quantification seems to be particularly relevant, given that their concentrations can vary by more than one order of magnitude under normal conditions.

When analyzing the differences between male and female samples, sex hormone-binding globulin (SHBG) was found to be higher in the latter. This result is in agreement with earlier studies [18,19], including the MRM result for the North American cohort [9], which also showed the higher concentrations of SHBG in female samples of Black and Hispanic groups. The significantly increased level of immunoglobulin heavy constant mu (IGHM, P01871) in female samples in this study, as well as significantly elevated apolipoprotein C-III (APOC3) in male samples, are also consistent with the data for the North American cohort, although their fold changes were slightly less than 1.5 times (Table 1).

Further comparison of the obtained data with the results for the North American cohort revealed 42 significantly differed proteins, including 24 FDA/CVD markers. These proteins are involved in interrelated immune, inflammation, coagulation, and complement pathways, which are of key importance in the development of vascular dysfunctions. These results may indicate the difference in the roles of specific (patho)physiological mechanisms in the development of a wide range of socially significant pathologies in North American and European cohorts. Particularly, in Europeans, the increased platelet activation, adhesion to the endothelium, activation of the plasma coagulation cascade, and secretion of proinflammatory cytokines may play the most important roles. In North Americans, socially significant diseases can be influenced by chronic inflammatory processes, impaired immune responses, and lipid metabolism.

According to the DESEASES database in the String web resource, of forty-two significantly different proteins, eight proteins are related to amyloidosis, eight to blood coagulation diseases, five to thrombophilia, thirteen to metabolic diseases, seven to kidney diseases, etc. The greatest difference was found for C-reactive protein (CRP, 4.5 times higher in North Americans) and P-selectin (SELPLG, 7 times higher in Europeans). C-reactive protein (CRP) deserves further discussion as a well-known CVD marker that has been widely studied in various ethnic cohorts. It is an acute-phase protein that increases following interleukin-6 and other cytokines’ secretion, and then it activates the complement. Kelley-Hedgepeth et al., when studying samples of women of different ethnicities without CVD (African American, Hispanic, Chinese, and Japanese), revealed the highest CRP levels in the African American group, while in Japanese and Chinese groups its content was minimal [20]. This relationship persisted even after adjusting for body mass index (BMI), age, socioeconomic status, and other risk factors, although it became less pronounced. It is noteworthy that the content of CRP in Europeans revealed in the current study (17 fmol/µL) turned out to be comparable to the levels in the Japanese group (20 fmol/µL); in North Americans (75 fmol/µL, according to Gaither et al. [9]), the average concentration of CRP is close to the level of CRP in the Hispanic group (92 fmol/µL, according to Kelley-Hedgepeth et al. [20]). Such coincidences further emphasize the high accuracy of the MRM-MS/MS method and its comparability to other quantitative methods. In addition to its reduced levels in the study cohort, C-reactive protein also showed the most variable concentrations, with a 17-fold change. So, its variability in different cohorts still requires reliable confirmation in a larger number of samples, and it should be considered in validation studies.

At the same time, significant intergroup differences in low-variable proteins may actually be due to different climatic and geographical living conditions, as well as ethnic and cultural affiliation. In particular, P-selectin, with concentration ranges of <5-fold, demonstrates the highest fold change between the samples of European and North American groups. P-selectin is a cell adhesion molecule that enhances the procoagulant activity and activation of leukocyte integrins. Its increased level can be a biomarker of platelet and endothelial activation, and it contributes to the development of atherosclerosis. The variability in the average level of P-selectin in different ethnic groups has already been noted previously [21].

Regarding the most common cardiovascular pathologies worldwide, certain associations with differences in lipid metabolism in different ethnic groups have previously been identified [22]. In the current study, apolipoproteins B100 and L1 (APOB, APOL1) turned out to be significantly higher in North Americans, while apolipoproteins C-I and F (APOC1, APOF) were higher in Europeans. It has also been shown that Asian Indians have even higher levels of APOB than Americans [23]. APOB is an identifier of increased risk of atherosclerotic CVD. APOL1 may play a role in the inflammatory response; furthermore, proinflammatory cytokines interferon-γ (IFNG), tumor necrosis factor-α (TNF-α), and p53 can enhance its expression [24]. Certain alleles of the APOL1 gene are associated with increased susceptibility to non-diabetic kidney disease. At the same time, APOF and APOC1, elevated in the European cohort, modulate lipoprotein metabolism by inhibiting the activity of cholesterol ester transfer protein, which exhibits a proatherogenic effect. However, there is increasing evidence of the proatherogenicity of APOC1, including its association with foam cell formation [25]. A recent study comparing different ethnic groups showed that APOC1 was lower in African Americans, Chinese Americans, and Hispanics vs. Whites, and positively associated with plasma triglycerides and high-density lipoproteins (HDL) [26], which is consistent with results of the current research.

Additionally, our study revealed that Europeans had elevated antigen-like CD5 (CD5L), a secreted protein that is also directly related to lipid metabolism as a key regulator of lipid synthesis. It is mainly expressed by macrophages in lymphoid and inflamed tissues, and regulates the mechanisms of inflammatory reactions, such as infection or atherosclerosis. Thus, the prevalence of one or another participant in lipid metabolism, on the one hand, may be due to characteristic diets, lifestyle, and genetic features, but also may reflect differences in the mechanisms of development of pathological processes. Although high HDL levels are thought to be atheroprotective, they do not protect against CVD in some ethnic groups (e.g., Native Americans) [27].

Differences between ethnic groups in the mechanisms of development of the same diseases have been described previously. It has been shown that a risk of different diseases may vary significantly, but the clinical factors of these circumstances is poorly understood. Thus, the results of Sjaarda et al. point to a strong role of ethnicity in insulin resistance and diabetes risk [28]. Many studies demonstrate the effect of ancestry on biomarker levels, suggesting that some of the observed differences in disease prevalence have a biological basis, and that reference intervals for these biomarkers should be adapted. A certain influence of genetic factors has been identified for C-peptide, apolipoprotein-E, intercellular adhesion molecule 1, eotaxin-3, clusterin, Fas ligand, fatty acid-binding protein liver, alpha-2-macroglobulin, interleukin 2, apolipoprotein-IV, and paraoxonase-2 in a study of 237 cardiometabolic markers in Latin Americans. Their significance was confirmed even after adjusting for smoking status, BMI, LDL-cholesterol levels, and fasting plasma glucose, in addition to age and sex [28].

Thus, the results of many studies strongly suggest that plasma concentrations of clinical biomarkers need to be calibrated for genetic and epigenetic factors, which is essential for the effective design of blood-based biomarker studies.

## 5. Conclusions

The performed MRM-MS analysis of samples from healthy volunteers of the European cohort revealed major agreement with the data obtained from 20 samples of a North American cohort that was analyzed in a similar MRM study using the same proteomic panel. The analysis revealed 15 significantly different proteins between male and female groups, and particularly confirmed higher levels of sex hormone-binding globulin (SHBG) and immunoglobulin heavy constant mu (IGHM) in females, as well as increased concentration of apolipoprotein C-III (APOC3) in males, also shown in the North American cohort. On the other hand, the most significant differences between the cohorts of the two continents were observed in the levels of 42 plasma proteins, mainly linked with the immune system and blood coagulation. This suggests that living conditions and lifestyle differences may have a more significant influence on the regulation of (patho)physiological pathways than genetic characteristics.

The results confirm the high reliability, efficiency, and reproducibility of MRM analysis based on “ready to use” kits, such as the BAK 270 assay. The used panel can serve as a basis for identifying protein marker panels for early recognition of the risk of developing specific pathologies. We believe that this approach is well-suited both for identifying marker panels for specific pathological conditions and for validating potential markers identified by other methods. The results obtained in this study, together with earlier data for North Americans, highlight the need for larger cohort studies on healthy donors to better understand and clarify the cause-and-effect relationships of various factors, leading to the development of socially significant pathologies.

## Figures and Tables

**Table 1:** Significantly different plasma proteins between male and female groups.

Protein Name	Code	UniProt ID	Medians Fold Change ^a^	*p*-Value
Apolipoprotein C-III	APOC3	P02656	1.45	0.005
Biotinidase	BTD	P43251	1.28	0.005
Cholinesterase	BCHE	P06276	1.38	0.008
Coagulation factor X	F10	P00742	1.23	0.001
Fibulin-1	FBLN1	P23142	0.78	0.003
Ig mu chain C region	IGHM	P01871	0.71	0.008
Peroxiredoxin-2	PRDX2	P32119	1.50	0.008
Phosphatidylinositol-glycan-specific phospholipase D	GPLD1	P80108	1.40	<0.001
Plasminogen	PLG	P00747	1.24	0.003
Retinol-binding protein 4	RBP4	P02753	**1.52** ^b^	<0.001
Serum amyloid P-component	APCS	P02743	**1.95**	<0.001
Sex hormone-binding globulin	SHBG	P04278	**0.53**	<0.001
Transthyretin	TTR	P02766	**2.13**	0.006
Vitamin K-dependent protein C	PROC	P04070	1.19	0.009
Vitamin K-dependent protein S	PROS1	P07225	1.31	0.001

^a^ The comparison of the median values determined for the male subgroup vs. the female subgroup; ^b^ fold differences of >1.5 or <0.67 are highlighted in bold.

**Table 2:** Significantly different plasma proteins between North American and European cohorts.

Protein Name	Code	UniProt ID	Medians Fold Change ^a^	*p*-Value
Alpha-1-antichymotrypsin	SERPINA3	P01011	1.15	<0.001
Alpha-1B-glycoprotein	A1BG	P04217	1.25	<0.001
Alpha-2-HS-glycoprotein ^b^	AHSG	P02765	1.47	<0.001
Antithrombin-III ^b,c^	SERPINC1	P01008	1.21	<0.001
Apolipoprotein B-100 ^b,c,d^	APOB	P04114	**1.91** ^e^	<0.001
Apolipoprotein C-I ^c,f^	APOC1	P02654	0.68	0.004
Apolipoprotein F	APOF	Q13790	**0.51**	<0.001
Apolipoprotein L1 ^c^	APOL1	O14791	1.31	0.007
Beta-2-microglobulin ^b,d^	B2M	P61769	0.85	0.003
C4b-binding protein alpha chain	C4BPA	P04003	1.38	<0.001
Cartilage acidic protein 1	CRTAC1	Q9NQ79	**1.80**	<0.001
CD5 antigen-like	CD5L	O43866	**0.52**	<0.001
Complement C1q subcomponent subunit A ^b^	C1QA	P02745	0.80	0.001
Complement C1q subcomponent subunit B ^b^	C1QB	P02746	**1.71**	<0.001
Complement C1q subcomponent subunit C ^b^	C1QC	P02747	**2.49**	<0.001
Complement C1r subcomponent-like protein	C1RL	Q9NZP8	1.14	0.007
Complement C3 ^b,c,d^	CPAMD1	P01024	1.19	0.004
Complement C5 ^b^	CPAMD4	P01031	1.13	0.001
Complement component C9 ^f^	C9	P02748	**1.59**	<0.001
Complement factor I	F1	P05156	1.44	<0.001
C-reactive protein ^b,c,d^	CRP	P02741	**4.52**	<0.001
Cystatin-C ^b^	CST3	P01034	0.79	<0.001
Extracellular matrix protein 1	ECM1	Q16610	0.86	0.007
Fibrinogen alpha chain ^b,d^	FGA	P02671	1.16	0.008
Fibrinogen beta chain ^b,d^	FGB	P02675	1.34	<0.001
Haptoglobin ^b,c,d,f^	HP	P00738	**1.97**	<0.001
Hemoglobin subunit alpha ^b^	HBG2	P69905	**0.26**	<0.001
Hemopexin ^b,c,d^	HPX	P02790	1.24	<0.001
Heparin cofactor 2	SERPIND1	P05546	1.33	0.008
Ig gamma-1 chain C region	IGHG1	P01857	**2.34**	<0.001
Immunoglobulin kappa variable 4-1 ^d^	IGKV4-1	P06312	**1.53**	<0.001
Kininogen-1	KNG1	P01042	1.18	<0.001
Lysozyme C	LYZ	P61626	1.42	<0.001
N-acetylmuramoyl-L-alanine amidase	AMIA	Q96PD5	1.09	0.008
Phospholipid transfer protein	PLTP	P55058	0.78	<0.001
Plasma protease C1 inhibitor ^d^	SERPING1	P05155	**1.82**	<0.001
P-selectin	SELPLG	P16109	**0.14**	<0.001
Retinol-binding protein 4	RBP4	P02753	**1.66**	<0.001
Serotransferrin ^d^	TF	P02787	1.19	<0.001
Tetranectin	TNA	P05452	**0.65**	<0.001
Vitamin K-dependent protein S	PROS1	P07225	1.15	0.01
Zinc-alpha-2-glycoprotein	AZGP1	P25311	**3.91**	<0.001

^a^ The comparison of the median values determined for North American [9] vs. European cohort. ^b^ FDA-approved markers; ^c^ markers of cardiovascular disease (CVD); ^d^ potential markers of Alzheimer’s disease; ^e^ fold differences of >1.5 or <0.67 are highlighted in bold; ^f^ potential oncomarkers.

## Data Availability

All experimental results were uploaded to the PeptideAtlas SRM Experiment Library (PASSEL), and are available via the following link: http://www.peptideatlas.org/PASS/PASS05887 (accessed on 28 August 2024).

## Supplementary Materials

The following supporting information can be downloaded at: https://www.mdpi.com/article/10.3390/biomedicines12102403/s1, Table S1: Calibration curve; Table S2: Calculated protein concentration in samples; Table S3: Concentrations of proteins in Quality Control (QC) samples; Table S4: Mean concentrations of proteins for 21 individuals and technical CV (%); Table S5: Mean, median and fold change of protein concentration (fmol/uL) in the 21 healthy Europeans; Table S6: Significantly changed proteins between males and females; Table S7: Significantly enriched processes for plasma proteins with different level between North American and European (current study) cohorts; Table S8: MRM transitions list for NAT/SIS peptides (Light/Heavy) from BAK270 kit; Table S9: A representative sample worklist.
